# An Effective Hypoxia-Related Long Non-Coding RNA Assessment Model for Prognosis of Lung Adenocarcinoma

**DOI:** 10.3389/fgene.2022.768971

**Published:** 2022-03-16

**Authors:** Yuanshuai Li, Xiaofang Sun

**Affiliations:** ^1^ Department of Obstetrics and Gynecology, Key Laboratory for Major Obstetric Diseases of Guangdong Province, The Third Affiliated Hospital of Guangzhou Medical University, Guangzhou, China; ^2^ Key Laboratory of Reproduction and Genetics of Guangdong Higher Education Institutes, Guangzhou, China

**Keywords:** lung adenocarcinoma, hypoxia-related prognostic signature, immune infiltrates, long non-coding RNAs, nomogram

## Abstract

**Background:** Lung adenocarcinoma (LUAD) represents one of the highest incidence rates worldwide. Hypoxia is a significant biomarker associated with poor prognosis of LUAD. However, there are no definitive markers of hypoxia-related long non-coding RNAs (lncRNAs) in LUAD.

**Methods:** From The Cancer Genome Atlas (TCGA) and the Molecular Signatures Database (MSigDB), we acquired the expression of hypoxia-related lncRNAs and corresponding clinical information of LUAD patients. The hypoxia-related prognostic model was constructed by univariable COX regression analysis, least absolute shrinkage and selection operator (LASSO), and multivariable Cox regression analysis. To assess the performance of the model, the Kaplan–Meier (KM) survival and receiver operating characteristic (ROC) curve analyses were performed.

**Results:** We found seven lncRNAs, AC022613.1, AC026355.1, GSEC, LINC00941, NKILA, HSPC324, and MYO16-AS1, as biomarkers of the potential hypoxia-related prognostic signature. In the low-risk group, patients had a better overall survival (OS). In addition, the results of ROC analysis indicated that the risk score predicted LUAD prognosis exactly. Furthermore, combining the expression of lncRNAs with clinical features, two predictive nomograms were constructed, which could accurately predict OS and had high clinical application value.

**Conclusion:** In summary, the seven-lncRNA prognostic signature related to hypoxia might be useful in predicting clinical outcomes and provided new molecular targets for the research of LUAD patients.

## Introduction

LUAD is the most common pathological subtype in lung cancer, accounting for 40% of all lung cancer incidences (Lemjabbar-Alaoui et al., 2015; Shi et al., 2016). The mean 5-year survival rate of patients was only 15% (Odintsov et al., 2021). The major reason for the high mortality of LUAD is that LUAD is diagnosed at an advanced stage in most patients. Currently, the diagnosis of LUAD is primarily based on symptoms, which included the size and location of the tumor, the location of the tumor in the lymph nodes, and where the cancer has spread (Lemjabbar-Alaoui et al., 2015; Rami-Porta et al., 2017; Carter et al., 2018). Although many potential biomarkers for early detection of LUAD have been studied, such as autophagy-related survival model, immune-related survival model, and ferroptosis-related gene signature, there is still lack of clinically used biomarkers due to lack of sensitivity and validity of these biomarkers on the development of LUAD (Hirsch et al., 2017; Chen et al., 2021; Shi et al., 2021; Wu et al., 2021; Jiang et al., 2021; Li et al., 2021).

As a non–protein-coding RNA, lncRNA has approximately 200 nucleotides in length, and it has attracted much attention because of its ability to regulate gene expression in epigenetic, transcriptional, and posttranscriptional dimensions (Chang et al., 2016; Fang and Fullwood, 2016; Li et al., 2016; Bhan et al., 2017; Peng et al., 2017). LncRNAs significantly affected the development of tumors (Chang et al., 2016; Choudhry et al., 2016). Recently, lncRNA-related prognostic models have been extensively developed for many cancers, including gastric cancer, lung cancer, pancreatic cancer, breast cancer, and colorectal cancer (Guo et al., 2020; Wang et al., 2020; Zhang H et al., 2021).

Hypoxia is one of the main characteristics of the tumor microenvironment (TME) and usually associated with poor prognosis. According to the study, many lncRNAs play a regulatory role in the hypoxia of tumors, such as participating in the regulation of tumor growth, vascular formation, invasion, and metastasis. Under hypoxic conditions, the lncRNA HABON could promote growth and proliferation of hepatocarcinoma cells (Ma C et al., 2021; Ma T et al., 2021). In the hypoxic environment of gastric cancer, the expression of the lncRNA LINC00460 is upregulated and promotes tumor invasiveness (Chen et al., 2020). The hypoxia-regulated lncRNA H19 and PDK1 (pyruvate dehydrogenase kinase 1) expression exhibits strong correlations in primary breast carcinomas, and they promote reprogramming of cancer stem cells (Peng et al., 2018). However, there are no exact prognostic markers related to hypoxia-related lncRNAs in LUAD.

In this study, we identified seven hypoxia-related lncRNAs strongly associated with OS. Meanwhile, a risk signature was constructed. By using the other cohort, the accuracy and reliability of this model were validated. Moreover, we found that the signature was independent of clinical features. In conclusion, we successfully established a risk model associated with hypoxia. Moreover, it may be used for clinical treatment and diagnosis.

## Materials and Methods

### Ethics Statement

**FIGURE 1:**
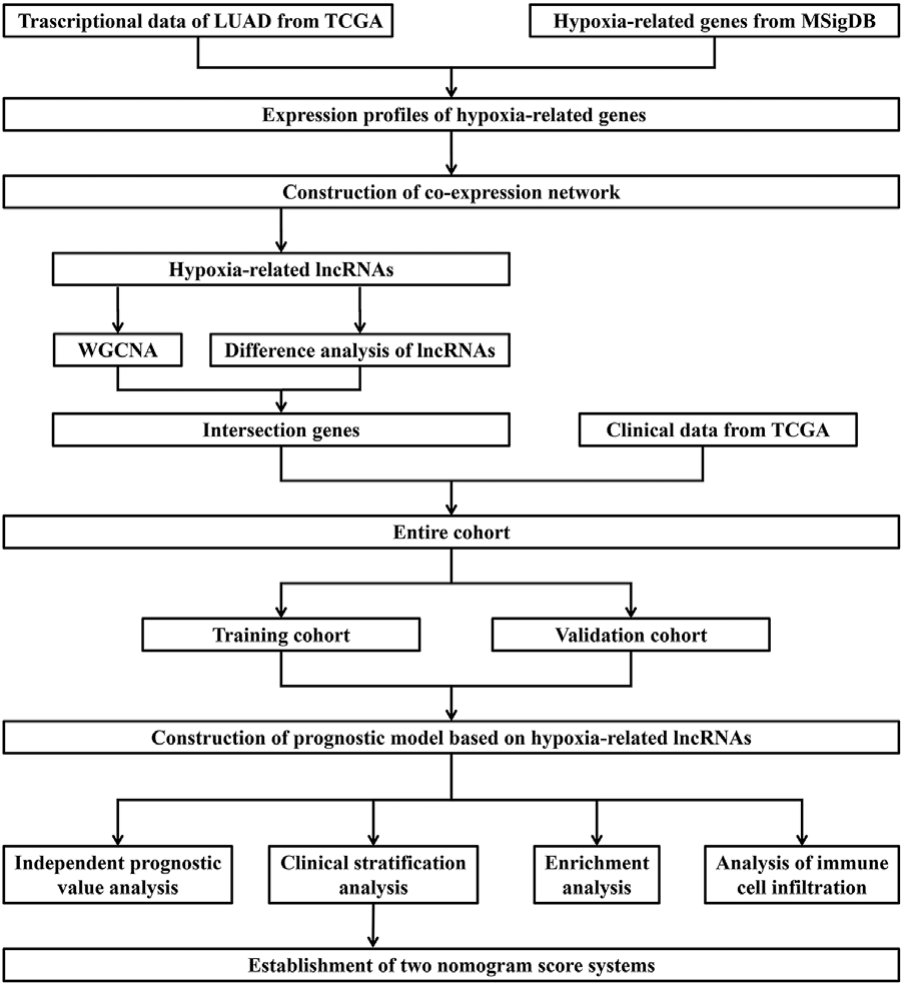
Flow chart of data acquisition and analysis.

The RNA-sequencing and clinical data of LUAD were downloaded from TCGA database (https://cancergenome.nih.gov/). Our study was based on the open resource data that were free for researching and publishing relevant articles with no ethical issues and other conflicts of interest. The process of this study is presented in Figure 1.

### Data Acquirement of TCGA

The mRNA expression data were derived from 535 LUAD patients and 59 healthy controls. Meanwhile, corresponding clinico-pathological data, including gender; age; pathologic T, M, and N stage; tumor clinical stage; and overall survival (OS) time were also obtained from TCGA database. Twenty-three out of 522 patients were excluded due to lack of information of OS (or the OS time was zero); therefore, lncRNA expression of 499 patients and their clinico-pathologic data were used for analysis. We presented the basic clinical information of these patients in Table 1.

**TABLE 1:** Basic clinical information of 499 LUAD patients from TCGA.

Variables	LUAD patients (N = 499)
Gender	
Female	270 (54%)
Male	229 (46%)
Age	
≤65 years	240 (48%)
>65 years	259 (52%)
Pathologic T Stage	
T1	170 (34%)
T2	263 (53%)
T3	45 (9%)
T4	18 (4%)
Unknown	3 (0%)
Pathologic M Stage	
M0	331 (66%)
M1	24 (5%)
Unknown	144 (29%)
Pathologic N Stage	
N0	322 (65%)
N1	95 (19%)
N2	69 (14%)
N3	2 (0%)
Unknown	11 (2%)
Pathologic Stage	
Stage I	266 (53%)
Stage II	120 (24%)
Stage III	80 (16%)
Stage IV	25 (5%)
Unknown	8 (2%)

Note: T, tumor size; M, distant metastasis; N, lymph node.

### Hypoxia-Related LnRNA Extraction

We performed Gene Set Enrichment Analysis (GSEA) to research two datasets associated with hypoxia (HARRIS_HYPOXIA, WINTER_HYPOXIA_METAGENE) from the MsigDB database. Then, the genes in all samples were analyzed in the abovementioned gene sets. There were 239 hypoxia-related genes found from the statistically significant gene set. Finally, by Pearson’s correlation analysis, we identified hypoxia-related lncRNAs with the criteria of |correlation coefficient| > 0.3 and *p*-value < 0.001 and then constructed an mRNA–lncRNA coexpression network connected with hypoxia.

### Identification of Hypoxia-Related LncRNAs

The Wilcoxon test was utilized to screen the differential expressed lncRNAs (DELs) between tumor and normal samples. The genes with *p*-value < 0.05 and |log_2_ fold-change (FC)| > 1 were defined as DELs. Then, we utilized the R package “WGCNA” to construct a scale-free coexpression network for the all hypoxia-related lncRNAs by setting the soft threshold power value to 4. Finally, we selected two models highly correlated with cancer samples for followed analysis.

### Construction and Validation of the Hypoxia-Related LncRNA Prognostic Signature

We first took the intersection of 601 DELs and 617 lncRNAs in the two modules (blue: 394, brown: 223). By using univariate Cox analysis, survival-related lncRNAs associated with hypoxia were identified. Then, LASSO regression analysis was used to further screen genes. At this time, we randomly divided the LUAD samples into two cohorts, training and validation cohorts. Finally, the lncRNAs were selected to construct a multivariate Cox regression model, and the risk score was calculated. Based on the median score of the training cohort, the patients were divided into high- and low-risk subgroups. Between the two groups, KM analysis was used to compare the survival time, and the ROC curve was used to evaluate the predictive power of the signature. In this way, the prognostic signature was constructed. In addition, in the other cohort, we performed the same procedure to evaluate the correctness of it.

### Functional Enrichment Analysis

We utilized GSEA v4.1 (http://www.gsea-msigdb.org/gsea/index.jsp) to perform GSEA between the low- and high-risk groups. After that, we carried out the Gene Ontology (GO) and Kyoto Encyclopedia of Genes and Genomes (KEGG) analyses of the differentially expressed mRNAs (DEGs) between the two groups, using “limma” and “clusterProfiler” packages.

### Correlation Analysis of the Tumor Microenvironment in 33 the Cancer Genome Atlas Pan-Cancers

We then downloaded 33 cancer types from the UCSC Xena database (https://xenabrowser.net/datapages/), and they are adrenocortical carcinoma (ACC), bladder urothelial carcinoma (BLCA), breast invasive carcinoma (BRCA), esophageal carcinoma (ESCA), cervical squamous cell carcinoma and endocervical adenocarcinoma (CESC), colon adenocarcinoma (COAD), lymphoid neoplasm diffuse large B-cell lymphoma (DLBC), glioblastoma multiforme (GBM), head and neck squamous cell carcinoma (HNSC), kidney chromophobe (KICH), kidney renal clear cell carcinoma (KIRC), kidney renal papillary cell carcinoma (KIRP), acute myeloid leukemia (LAML), brain lower grade glioma (LGG), liver hepatocellular carcinoma (LIHC), lung adenocarcinoma (LUAD), lung squamous cell carcinoma (LUSC), mesothelioma (MESO), ovarian serous cystadenocarcinoma (OV), pancreatic adenocarcinoma (PAAD), pheochromocytoma and paraganglioma (PCPG), prostate adenocarcinoma (PRAD), rectum adenocarcinoma (READ), sarcoma (SARC), skin cutaneous melanoma (SKCM), testicular germ cell tumors (TGCT), thyroid carcinoma (THCA), thymoma (THYM), uterine corpus endometrial carcinoma (UCEC), uterine carcinosarcoma (UCS), cholangiocarcinoma (CHOL), stomach adenocarcinoma (STAD), and uveal melanoma (UVM). Meanwhile, six types of immune infiltration, namely, C1 (wound healing), C2 (INF-r dominant), C3 (inflammatory), C4 (lymphocyte depleted), C5 (immunologically quiet), and C6 (TGF-β dominant) were also downloaded from it. Spearman’s analysis was used to calculate the association of the lncRNAs with immune subtypes, tumor mutation burden (TMB), and stemness score (RNAss and DNAss).

### Statistical Analysis

All statistical analyses were accomplished with R software (version 4.0.5). The DEGs were identified by the Wilcoxon test. We used Pearson’s correlation analysis to calculate the correlation between lncRNAs and mRNAs associated with hypoxia. Meanwhile, we applied univariate and multivariate Cox regression analyses to evaluate the correlation between the risk score and clinical features. *P*-value < 0.05 was regarded as a significant outcome.

## Results

### Identification of Hypoxia-Related LncRNAs in LUAD

**FIGURE 2:**
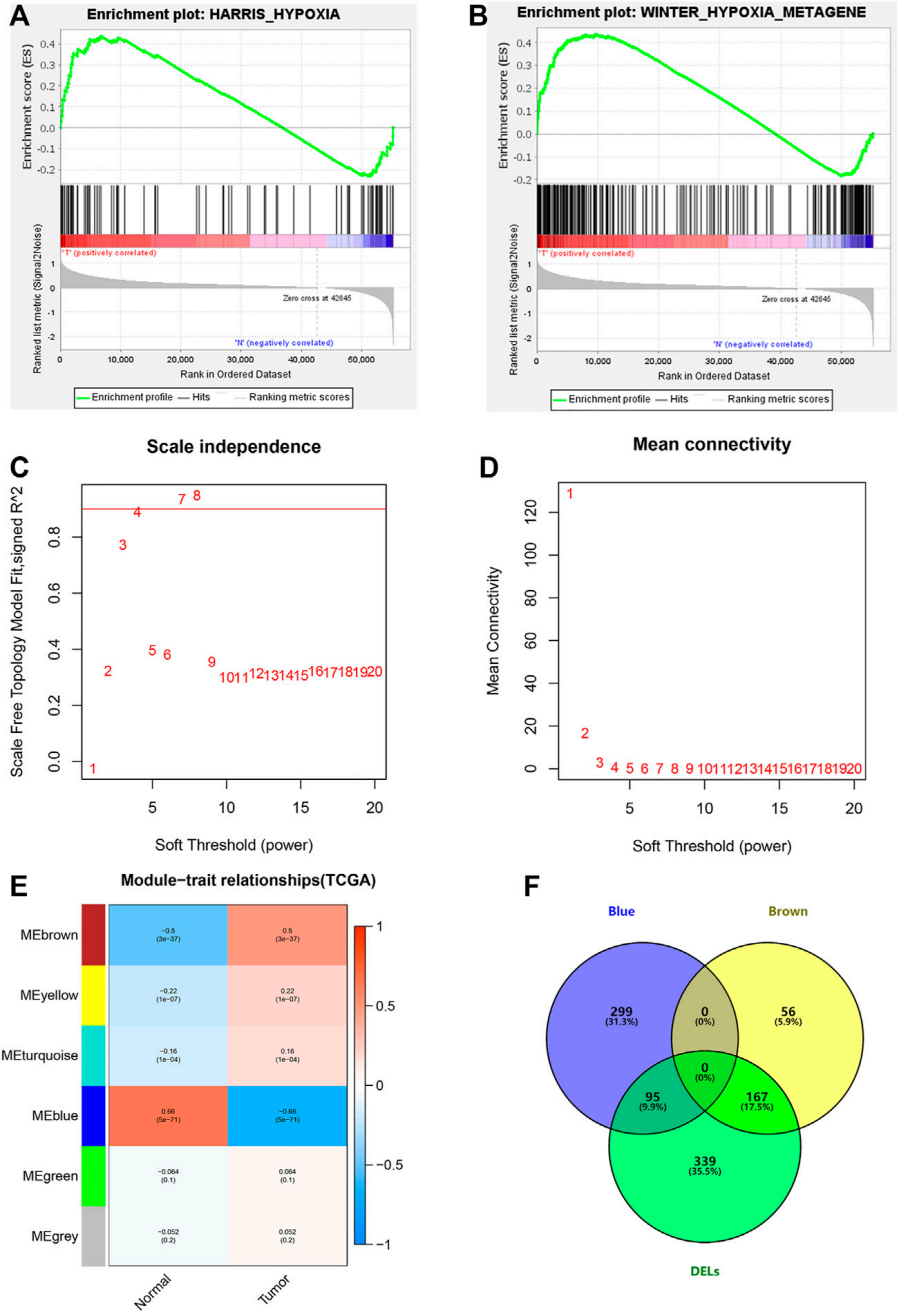
Hypoxia-related lncRNA extraction. **(A,B)** GSEA analysis showing the most enriched hypoxia-related pathways. **(C,D)** Determination of the most suitable power value for scale-free coexpression network. **(E)** Brown and blue modules were the most correlated with the tumor state. **(F)** Identification of common genes between DELs and the brown and the blue modules by overlapping them.

Through GSEA, we found that the WINTER_HYPOXIA_METAGEN gene set was significantly enriched [(FDR = 0.040), (Supplementary Table S1)], while the HARRIS_HYPOXIA gene set was not [(FDR = 0.164), (Figures 2A,B)]. Next, 1,629 hypoxia-related lncRNAs were screened according to the significant gene set (Supplementary Table S2).

We further studied hypoxia-related lncRNAs by the means of the Wilcoxon test and the weighted gene co-expression network (WGCNA) analysis, and we used the 601 differentially expressed hypoxia-related lncRNAs between normal and tumor samples to intersect with the 617 lncRNAs from the two modules of WGCNA analysis (Figures 2C–F).

### Construction of Hypoxia-Related LncRNA Prognostic Signature for LUAD

**FIGURE 3:**
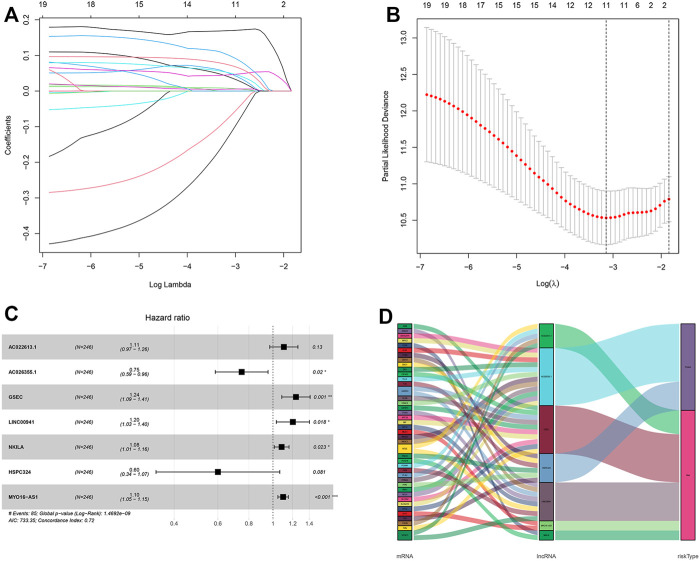
Identification and construction of a hypoxia-related lncRNA signature in the training cohort. **(A,B)** Robust lncRNAs were screened by LASSO analysis. **(C)** Forest plot of the multivariate Cox regression model. **(D)** Sankey diagram shows the lncRNA–mRNA interaction about hypoxia. * represents *p* < 0.05, ** represents *p* < 0.01, and *** represents *p* < 0.001.

After taking the intersection, we got 262 hypoxia-related lncRNAs (Supplementary Table S3). We then used these lncRNAs to construct the prognostic signature. First, 20 lncRNAs were screened by univariate Cox analysis [(*p*-value < 0.05), (Supplementary Table S4)]. Then, by using LASSO analysis, 20 variables were reduced to 11 potential predictors (Figures 3A,B). Finally, seven lncRNAs were identified by the multivariate Cox regression analysis in the training cohort. LncRNAs, AC022613.1, AC026355.1, GSEC, LINC00941, NKILA, HSPC324, and MYO16-AS1, were used to calculate the risk score (Figures 3C–E). Prognostic risk genes correlated with hypoxia were constructed (Table 2).

**TABLE 2:** Information of seven hypoxia-related lncRNAs associated with OS in patients with LUAD.

lncRNA symbol	Cox (β)	HR
AC022613.1	0.100829689	1.106088246
AC026355.1	−0.29010788	0.748182849
GSEC	0.213042416	1.237437137
LINC00941	0.184433915	1.202537509
NKILA	0.080054987	1.083346635
HSPC324	−0.510440682	0.600231009
MYO16-AS1	0.094137118	1.098710388

The unique risk score of patients was calculated through multivariate Cox analysis and the expression level. Risk score = expression of AC010980.2 × 0.490289217 + expression of AC026355.1 × −0.417505811 + expression of AL606489.1 × 0.293303854 + expression of ITGB1-DT × 0.289653582 + expression of AL034397.3 × −0.277395699 + expression of LINC01116 × 0.192509771 + expression of LINC01150 × −0.506323639. According to the median of the risk score, there were 123 patients in the high- and low-risk groups, respectively. Additionally, in the other cohort, the number was 130 and 114 patients using the same middle score.

**FIGURE 4:**
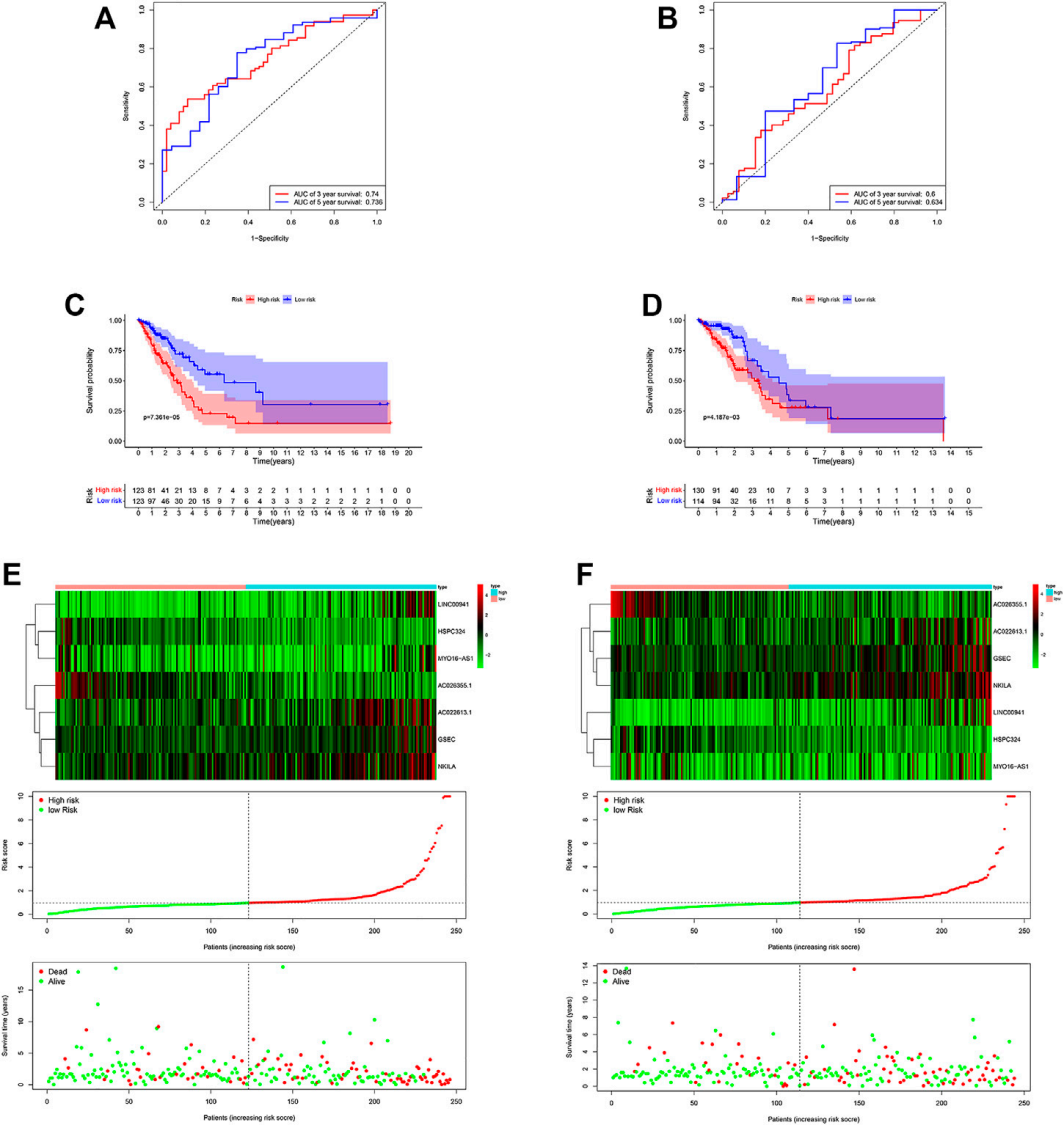
Risk score of the hypoxia-related lncRNA signature for survival prediction in the two cohorts. The ROC analysis showed that the signature was stable **(A)** in the training and **(B)** in the validation cohorts. Between low- and high-risk groups, KM analysis was used for comparison of the OS **(C)** in the training and **(D)** validation cohorts. Distributions of risk score, survival status, and expression of lncRNAs **(E)** in the training, and **(F)** validation cohorts.

By applying ROC curve analysis, in the training cohort, the area under the curve (AUC) was 0.740 at year 3 and 0.736 at year 5. KM analysis also showed that the model could be a valid prognostic indicator for patients (Figure 4A). Likewise, in the other cohort, the AUC values were 0.600 and 0.634, respectively (Figure 4B). From the result of KM analysis, we got the same trend with the training cohort (Figures 4C,D). Meanwhile, we found that in both cohorts, patients in the low-risk group had more survival time than those in the high-risk group (Figures 4E,F). In addition, in the training cohort, the result of the C-index was 0.715 and 0.643 in the other cohort.

### Independent Prognostic Value of the Signature

**FIGURE 5:**
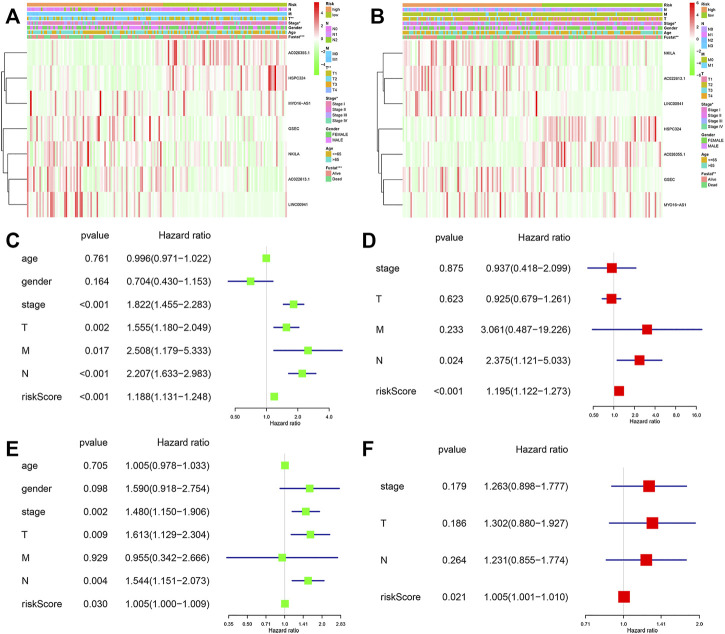
Forest plots of the univariate and multivariate Cox analysis in LUAD patients. Heatmap and clinico-pathologic features of the seven hypoxia-related lncRNAs **(A)** in the training, and **(B)** validation cohorts. Univariate and multivariate analyses for OS **(C,D)** in the training and **(E,F)** validation cohorts.

Among these lncRNAs, two lncRNAs in the training cohort (AC026355.1 and HSPC324) were upregulated, while MYO16−AS1, GSEC, NKILA, AC022613.1 and LINC00941 were downregulated in the low-risk group. In addition, similar results were obtained in the validation cohort (Figures 5A,B). Moreover, in both cohorts, we used univariate and multivariate Cox regression analyses to assess whether the risk score could serve as an independent prognostic factor. The risk score was an independent factor revealed by the univariate Cox regression, and the HR of it was 1.442. In the multivariate analysis, the risk score also remained an independent prognostic indicator [*p* < 0.001, HR = 1.391, 95% CI: 1.258–1.540 (Figures 5C,D)] in the training cohort. In the validation cohort, the risk score was an independent prognostic indicator, too (Figures 5E,F).

**FIGURE 6:**
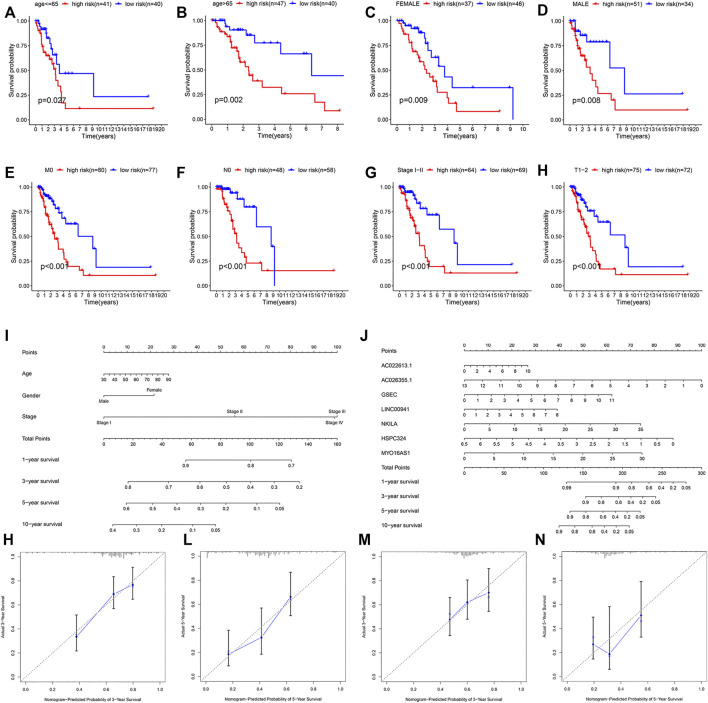
Stratification analysis of various clinico-pathological factors, nomograms used to predict the OS prognosis, and calibration plots used to predict prognosis in these patients with LUAD. KM curves of OS in the subgroups of **(A,B)** both the age groups, **(C,D)** both gender groups, **(E)** M0 group, **(F)** T0 group, **(G)** clinical stage I–II group, and **(H)** T1–2 group. **(I,J)** Nomograms to predict the 1-, 3-, 5-, and 10-year OS. Calibration plots for 3-year survival and 5-year survival **(K,L)** in the training and **(M,N)** validation cohorts.

In addition, to study the applicability of this model, we also conducted validations in different clinical subgroups. The patients were sorted by age (the 65 years or younger group and the more than 65 years group), gender (the male group and the female group), T stage of the tumor (the T1–T2 group and the T3–T4 group), M stage of the tumor (the M0 group and the M1 group), N stage of the tumor (the N0 group and the N1–N3 group), and tumor stage (the stage I–II group and the stage III–IV group). The results showed that the survival rate of high-risk patients with different age, gender, M0, N0, T1–T2, and stage I–II group was significantly different from that of low-risk patients (*p*-value < 0.05) in the training cohort (Figures 6A–H). In the validation cohort, we obtained the similar result (Supplementary Figure S1).

We then constructed two nomograms that integrated the risk score of the seven-lncRNA models and clinico-pathological features to predict survival probability of patients. Based on these, we predicted the patient’s 1-, 3-, 5-, and 10-year survival probabilities (Figures 6I,J). In both the nomograms, the higher the total points calculated, the worse the prognosis. Meanwhile, the calibration plot for the prediction of 3-year and 5-year survival also indicated the consistency between observation and prediction in both the cohorts (Figures 6K–N).

### Functional Analyses Based on the Risk Model

**FIGURE 7:**
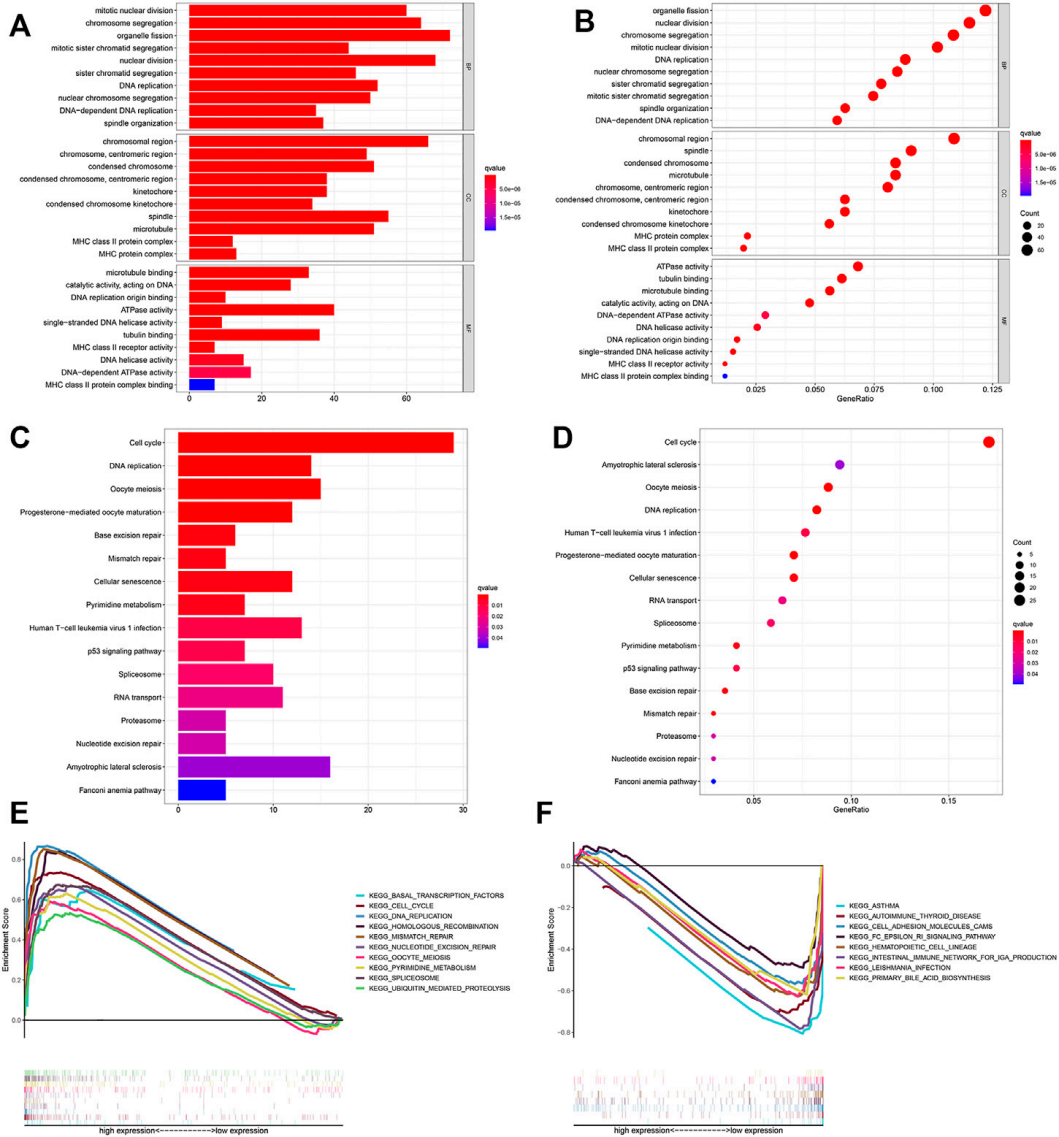
Functional analysis. Bubble and barplot graph for **(A,B)** KEGG pathways and **(C,D)** GO enrichment based on the DEGs in the training cohort. **(E,F)** KEGG pathways of GSEA analysis.

We used GSEA software to perform KEGG analysis for exploring which pathways were enriched. The results identified that in the high-risk group, processes such as cell cycle, DNA replication, and mismatch repair were enriched by using the training cohort (Figures 7A,B).

We further screened 660 different genes (adjust *p*-value < 0.05 and |log2FC (fold-change)| > 1) between the high- and low-risk groups and carried out enrichment analysis based on these in the training cohort. GO enrichment analysis indicated that many nuclear biological processes or molecular functions were significantly enriched (Figures 7C,D). Next, KEGG pathway analysis indicated that the biological processes related to cell proliferation were enriched, based on the upregulated genes, while downregulated genes were highly correlated with the immune process (Figures 7E,F and Supplementary Figures S2, S3). It was further verified that hypoxia is related to cell proliferation and immunity.

### Comparison of Immune Cell Infiltration Between Subgroups

**FIGURE 8:**
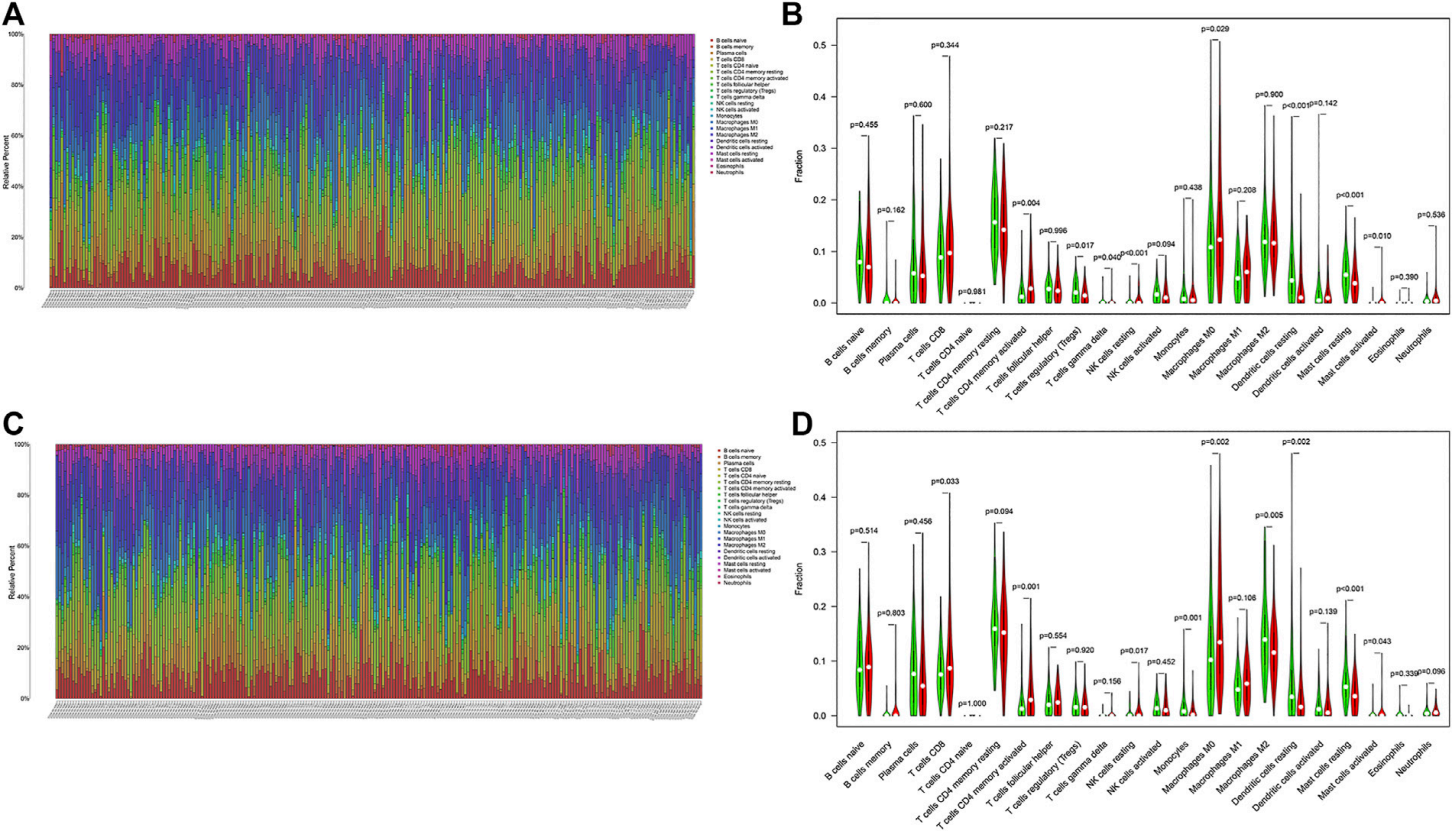
Landscape of immune cell infiltration in LUAD. Immune landscape of the patients with LUAD **(A)** in the training and **(C)** validation cohorts. Relationships between the risk score and immune cell infiltration **(B)** in the training and **(D)** validation cohorts. Red and green represent the high- and low-risk groups, respectively.

We calculated the proportion of 22 immune cells of all samples in the training and validation cohorts by using the CIBERSORT algorithm (Figures 8A,C). The violin plot showed that patients in the high-risk group had a higher proportion of activated memory CD4+ T cells, resting NK cells, M0 macrophages, and activated mast cells and a lower proportion of regulatory T cells, resting mast cells, and resting dendritic cells than those in the low-risk group in the training cohort (Figure 8B). In addition, in the validation cohort, CD8+ T cells, activated memory CD4+ T cells, resting NK cells, M0 macrophages, and activated mast cells were higher in the high-risk group than in the other group, whereas monocytes, M2 macrophages, resting mast cells, and resting dendritic cells were lower (Figure 8D). This result indicated that immune-related activities were associated with hypoxia.

**FIGURE 9:**
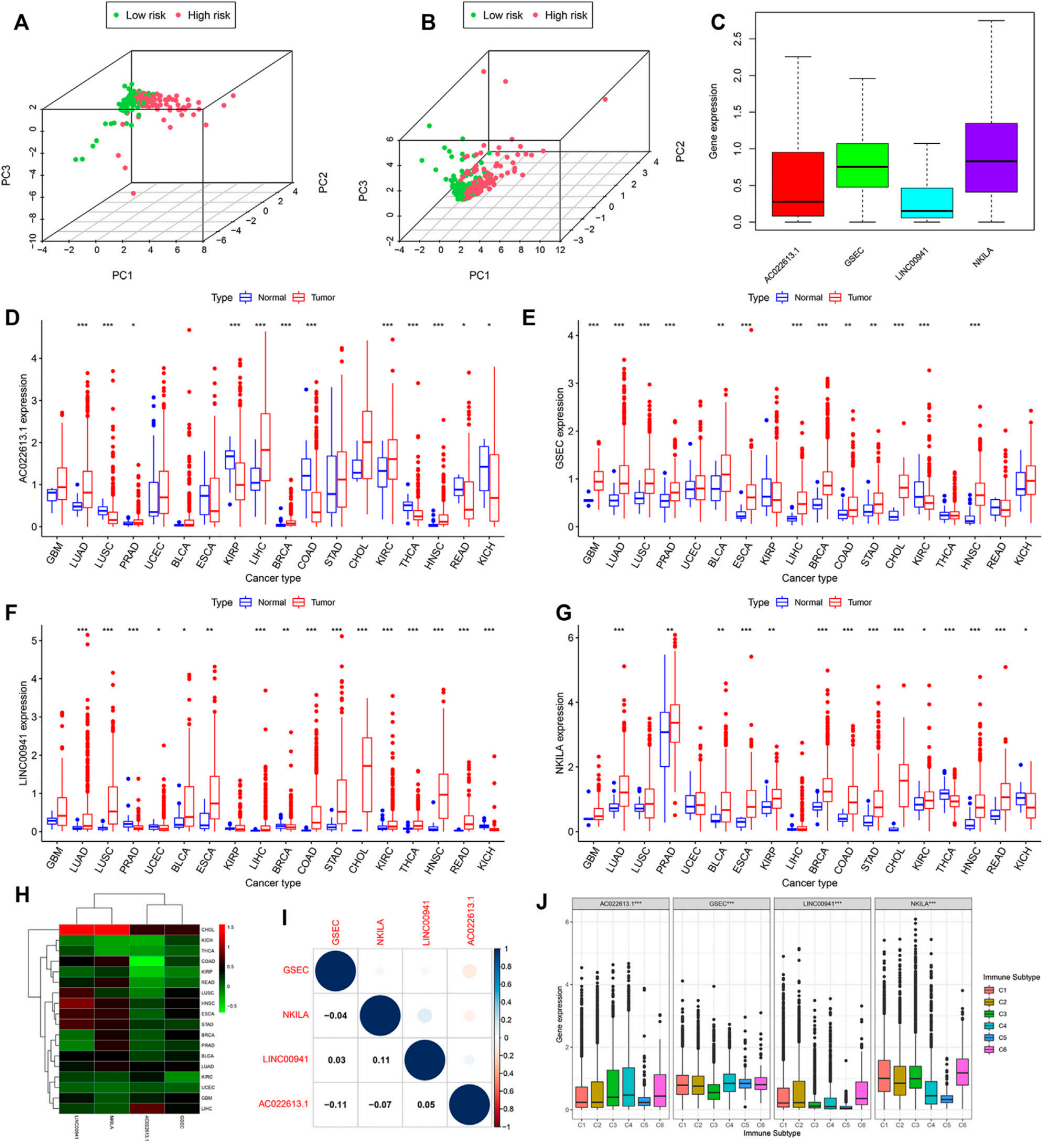
Expression of lncRNAs in pan-cancer which had more than five normal samples. Principal component analysis based on the lncRNAs of the model **(A)** in the training and **(B)** validation cohorts. **(C)** Expression of lncRNAs of the model is shown by the boxplot in pan-cancer. **(D–G)** In 18 cancer types, the difference of the expression of lncRNAs between tumor and normal samples is shown. **(H)** Heatmap showing the difference between normal and tumor samples of the expression of lncRNAs. **(I)** Relationship calculated by Spearman’s correlation analysis of the lncRNAs in pan-cancer. **(J)** Association tested by ANOVA in all cancers of four lncRNAs with six immune subtypes.

In addition, based on the prognostic signature, there was an observably different distribution between high- and low-risk groups through the principal component analysis, which indicated that there was a difference in the hypoxia phenotype of the model (Figures 9A,B).

### Association of Model LncRNAs With the Tumor Microenvironment and Immune Infiltration in Pan-Cancer

From the abovementioned results, the seven-model lncRNAs played an important role in LUAD. We then downloaded 33 cancer types to understand the function of the lncRNAs and selected AC022613.1, GSEC, LINC00941, and NKILA for further study. We found that AC022613.1, GSEC, LINC00941, and NKILA were mainly upregulated in tumor samples compared with normal samples (Figures 9D–G). In addition, the expression of these lncRNAs varied in different tumors (Figure 9C). The expressions of LINC00941 and NKILA were highly expressed in CHOL samples than those in the other tumors. In LUAD tissues, the expressions of the four lncRNAs were less than zero (Figure 9H). In addition, we found that LINC00941 and NKILA may have similar functions (Figure 9I).

**FIGURE 10:**
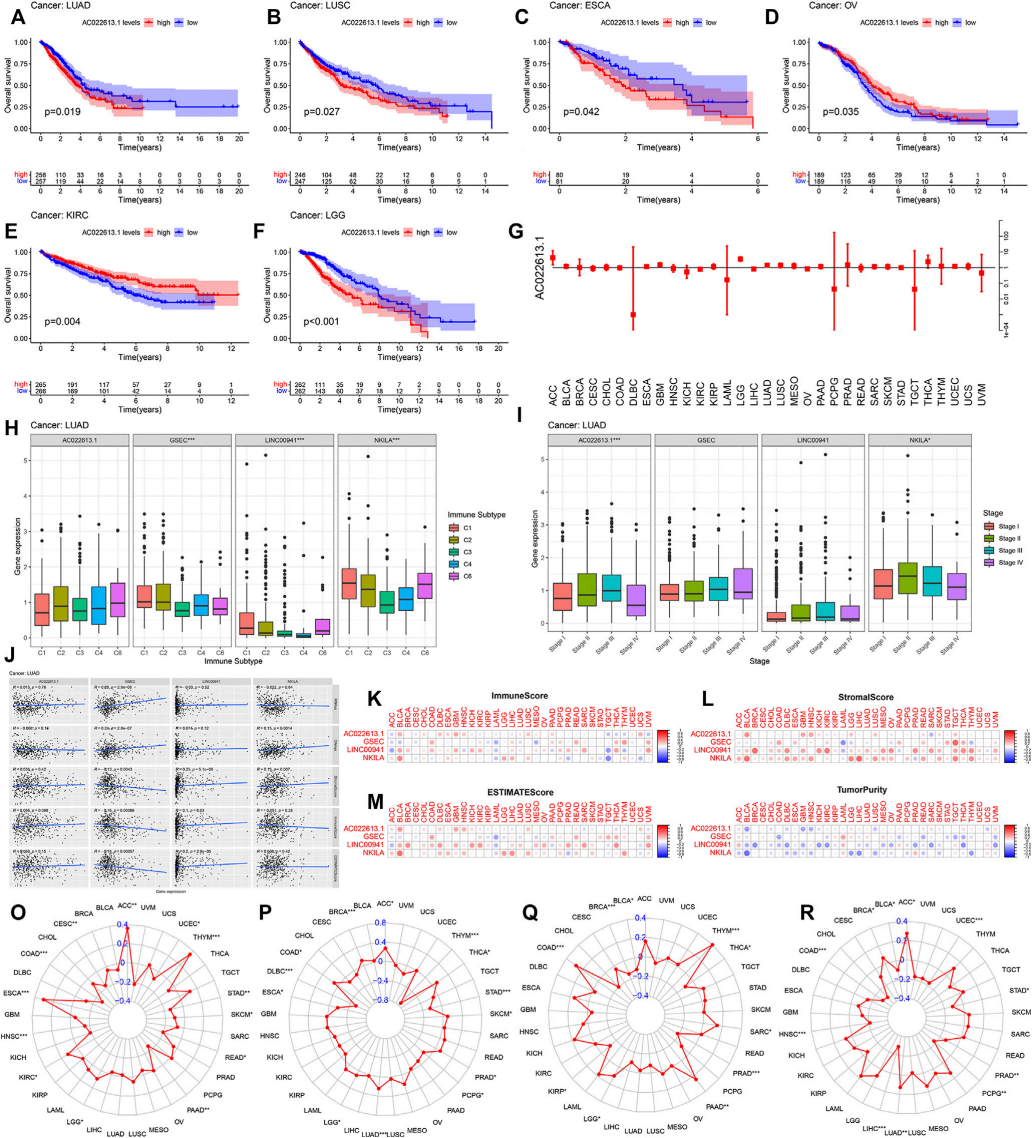
Correlation analysis between the four lncRNAs and patient prognosis and TMB in all cancer types. **(A–F)** Connection of the four lncRNAs with the prognosis of patients in pan-cancer. **(G)** Forest plot showing the hazard ratio of AC022613.1 across all cancer types. The correlation between the four lncRNAs and **(H)** immune subtypes and **(I)** clinical stage of LUAD. **(J)** Association of four lncRNAs with tumor stem cell scores and stromal, immune, and ESTIMATE score by Spearman’s correlation analysis. **(K–N)** The correlation relationship between four lncRNAs and stromal, immune, ESTIMATE, and tumor purity score. In 33 TCGA cancer types, the radar graph showing the association of the expression of **(O)** AC022613.1, **(P)** GSEC, **(Q)** LINC00941, and **(R)** NKILA with TMB. *p* < 0.05, *p* < 0.01, and *p* < 0.0001 were represented by *, **, and ***, respectively.

By plotting KM curves for 32 cancer types, we found that only AC022613.1 significantly affected the survival of patients in many cancer types (Figures 10A–F). Furthermore, we used univariate Cox analysis to investigate the relationship between the expression of AC022613.1 and patient survival. The result showed that the relationship was different in different tumors (Figure 10G). We then explored their role in the six immune subtypes, stromal, ESTIMATE, tumor purity, immune score, tumor stem cells, and TMB. In LUAD patients, we found that AC022613.1 was strongly connected with the tumor stage and GSEC, LINC00941, and NKILA were significantly connected with immune subtypes (Figures 10H,I). Based on the ESTIMATE analysis, we researched the connection between the four lncRNAs and tumor microenvironment in LUAD. The results showed that GSEC had a negative association with stromal, ESTIMATE, and immune score, while LINC00941 had the opposite result (Figure 10J). Meanwhile, the results indicated that in pan-cancer, the four lncRNAs were strongly associated with immune subtypes (Figure 9J). Moreover, we found that they were mainly positively connected with stromal, ESTIMATE, and immune score, while there was a negative correlation between these and tumor purity, RNAss, and DNAss in pan-cancer (Figures 10K–N). Furthermore, we used the radar plots to distribute their association between the four lncRNAs and TMB. Distinctly, we found that NKILA and GSEC had strongly correlation in LUAD patients (Figures 10O–R).

## Discussion

Overall, in this study, we obtained 239 hypoxia-related genes. According to the expression levels of the 239 genes, we identified 1,629 hypoxia-related lncRNAs using Pearson’s correlation analysis. |Correlation coefficient| > 0.3 and *p*-value < 0.001 were our selection criteria. In addition, there were many useful tools that could help extract hypoxia-related lncRNAs, such as BioSeq-BLM (Li et al., 2021), BioSeq-Analysis 2.0 (Liu et al., 2019), and starBase v2.0 (Li et al., 2014). However, they were mainly used for residue-level analysis and sequence-level analysis. In this study, according to the expression levels of the transcriptome, we used Pearson’s correlation analysis to identify lncRNAs closely associated with hypoxia. The correlation between the expression levels of mRNAs and lncRNAs was fully considered. Meanwhile, this method was widely used in the computational genomics field of tumors, such as hepatocellular carcinoma (Zhou et al., 2021), bladder cancer (Ma et al., 2021), soft tissue sarcomas (Zhang J et al., 2021), and breast cancer (Zhang L et al., 2021). There were 601 DELs associated with hypoxia between normal and tumor samples. Of them, 530 differentially expressed hypoxia-related lncRNAs were upregulated in the tumor samples, and 71 lncRNAs were downregulated. In addition, by performing WGCNA, we obtained 617 hypoxia-related lncRNAs that were associated with tumor samples. By taking the intersection of the 601 DELs related to hypoxia and 617 hypoxia-related lncRNAs, we got 262 hypoxia-related lncRNAs. Finally, we used univariate Cox analysis, LASSO analysis, and multivariate Cox analysis to generate the hypoxia-related lncRNA signature. We identified seven lncRNAs associated with hypoxia as potential prognostic biomarkers. KM analysis indicated that in the high-risk group, the OS of patients was shorter than that of patients in the low-risk group. Meanwhile, the seven-lncRNA signature was highly sensitive in the prediction of OS time of LUAD patients by taking the ROC analysis, and the results were further verified in the validation cohort. Finally, we constructed two nomograms to calculate a score representing the OS of LUAD patients.

In the signature, there were seven different lncRNAs in total. These lncRNAs were AC026355.1, AC022613.1, GSEC, LINC00941, NKILA, HSPC324, and MYO16-AS1. Among these hypoxia-related lncRNAs, according to reports, AC026355.1 is connected with the development of multiple tumors. It had important prognostic significance in both immune- and autophagy-related models (Li et al., 2020; You et al., 2021; Jiang et al., 2021). LINC00941 is one of the immune-related prognostic models comprising 7 lncRNAs in LUAD (Jin et al., 2020; Li et al., 2021). GSEC has only been described in osteosarcoma cells. In osteosarcoma cells, the overexpression of GSEC can enhance the proliferation and migration of tumor cells (Liu et al., 2020). LINC00941 usually was the risk factor and connected with worse survival (Chang et al., 2021; Fang et al., 2021; Wang et al., 2021). Wang Jie et al. found that in pancreatic cancer, LINC00941 was overexpressed and patients yielded worse prognosis (Wang et al., 2021; Chang et al., 2021). However, this lncRNA has not been reported in LUAD. NKILA is a tumor suppressor that affects the proliferation and metastasis of cancer cells by regulating the STAT3 pathway (Ashrafizadeh et al., 2021). LncRNA HSPC324 plays a crucial role in tumorigenesis of LUAD (Jafarzadeh et al., 2020). MYO16-AS1 was an oncogenic lncRNA in bladder cancer (Jafarzadeh et al., 2020). It has rarely been reported in LUAD. In conclusion, these lncRNAs all play a significant role in the occurrence and development of tumors.

A growing body of evidence suggests that the prognosis of tumor patients is connected with the level of immune invasion of the tumor, and the state of immune invasion is a key determinant of tumor development in the tumor microenvironment (Tao et al., 2021). Hypoxia of tumor tissue plays a vital role in promoting tumor immunosuppression and immunotherapy resistance. In this state, there are often abundant tumor-associated macrophages and Tregs, which inhibit the function of CD8+T cells and CD4+T cells (Dehghani et al., 2012; Sanchez-Martinez et al., 2018). Hypoxia inhibits the activity of effector T cells and NK cells, leading to decreased immune function. In our study CD8+ T cells; resting NK cells; M0, M1, and M2 macrophages; resting dendritic cells; and resting mast cells were found to be differentially infiltrated in LUAD and normal tissues, which is closely related to the development of tumors. This finding supported that the hypoxia-related lncRNA signatures reflected immune infiltration to some extent, providing meaningful information for immunotherapy (Kumar and Gabrilovich, 2014; Labiano et al., 2015; Aponte-Lopez and Munoz-Cruz, 2020).

## Conclusion

In summary, our study demonstrated that hypoxia is connected with the development of LUAD. Meanwhile, the two predictive nomograms were established for predicting the prognosis of LUAD patients. We anticipated that the study will provide an important basis for studies on the correlation between hypoxia-related genes and LUAD.

## Data Availability

The datasets presented in this study can be found in online repositories. The names of the repository/repositories and accession number(s) can be found below: Publicly available datasets were analyzed in this study. These data can be found here: The Cancer Genome Atlas (https://portal.gdc.cancer.gov/), the Molecular Signatures Database (https://immport.niaid.nih.gov), and CIBERSORT (https://cibersort.stanford.edu/).

## Abbreviations

C-index, c concordance index; DELs, differentially expressed lncRNAs; GO, Gene Ontology; GSEA, Gene Set Enrichment Analysis; KEGG, Kyoto Encyclopedia of Genes and Genomes; KM, Kaplan–Meier analysis; LUAD, lung adenocarcinoma; LASSO, least absolute shrinkage and selection operator; LncRNAs, long non-coding RNAs; MsigDB, The Molecular Signatures Database; OS, overall survival time; PCA, principal component analysis; ROC, receiver operating characteristic; TCGA, The Cancer Genome Atlas database; WGCNA, weighted gene co-expression network analysis.
