# Supplementary material for: An Effective Hypoxia-Related Long Non-Coding RNA Assessment Model for Prognosis of Lung Adenocarcinoma
Source: Front Genet. 2022 Mar 16;13:768971. doi: 10.3389/fgene.2022.768971 (PMC8966506; doi:10.3389/fgene.2022.768971)
Supplement: Supplementary file 1 [file DataSheet1.ZIP › Supplementary Material/Supplementary Material.docx]

Supplementary Material

# Supplementary Figures and Tables

## Supplementary Figures

**Supplementary Figure 1.** Stratification analysis of various clinicopathological factors for the patients with LUAD in the validation cohort. Kaplan-Meier curves of OS in the subgroups of (A) the age <= 65 group, (B) the male group, (C) the clinical stage III-IV group, and (D) the T3-4 group.

**Supplementary Figure 2.** Go enrichment analysis in the up-regulated and down-regulated differential genes of the traing cohort. Bubble, barplot, and circle graph for GO enrichment (A, B, C) in the up-regulated. (D, E, F) in the down-regulated.

**Supplementary Figure 3.** KEGG analysis based on the DEGs between the two-risk groups in the traing cohort. The KEGG analysis by barplot, bubble, and circle chart (A, B, E) in the up-regulated genes; (C, D, F) in the down-regulated genes.

## Supplementary Tables

**Supplementary Table 1.** The hypoxia-related mRNAs from the WINTER_HYPOXIA_METAGEN gene set.

**Supplementary Table 2.** The result of hypoxia-related lncRNAs identified by pearson correlation analysis.

**Supplementary Table 3.** The hypoxia-related lncRNAs identified by the intersection of 601 DELs and 617 lncRNAs in the two module (Blue: 394, Brown: 223).

**Supplementary Table 4.** The result of univariate Cox analysis in the all patients with LUAD.
