# Supplementary figures and images for: An Effective Hypoxia-Related Long Non-Coding RNA Assessment Model for Prognosis of Lung Adenocarcinoma
Source: Front Genet. 2022 Mar 16;13:768971. doi: 10.3389/fgene.2022.768971 (PMC8966506; doi:10.3389/fgene.2022.768971)

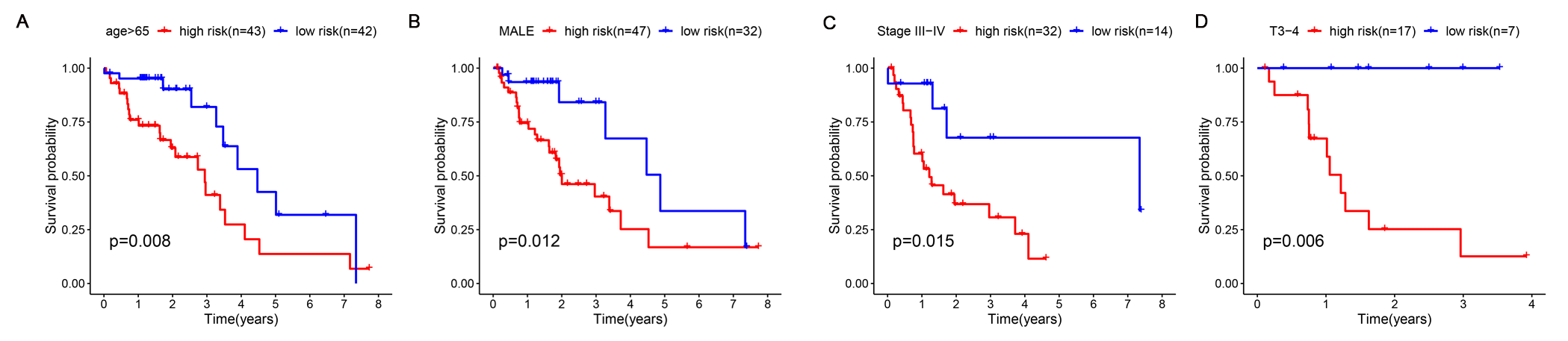

Supplement: Supplementary file 1 [file DataSheet1.ZIP › Supplementary Material/Supplementary Figure 1.tif]

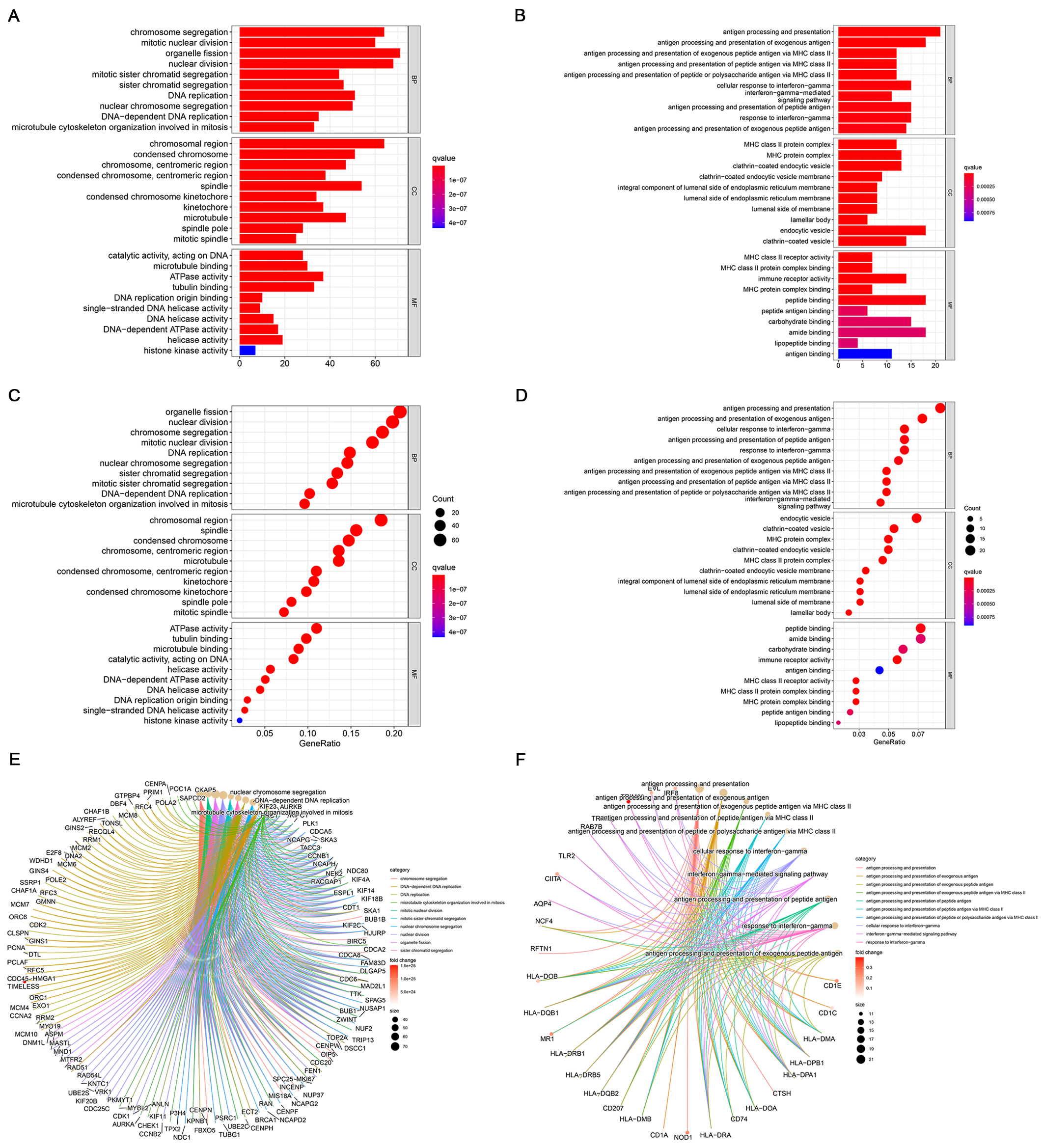

Supplement: Supplementary file 1 [file DataSheet1.ZIP › Supplementary Material/Supplementary Figure 2.tif]

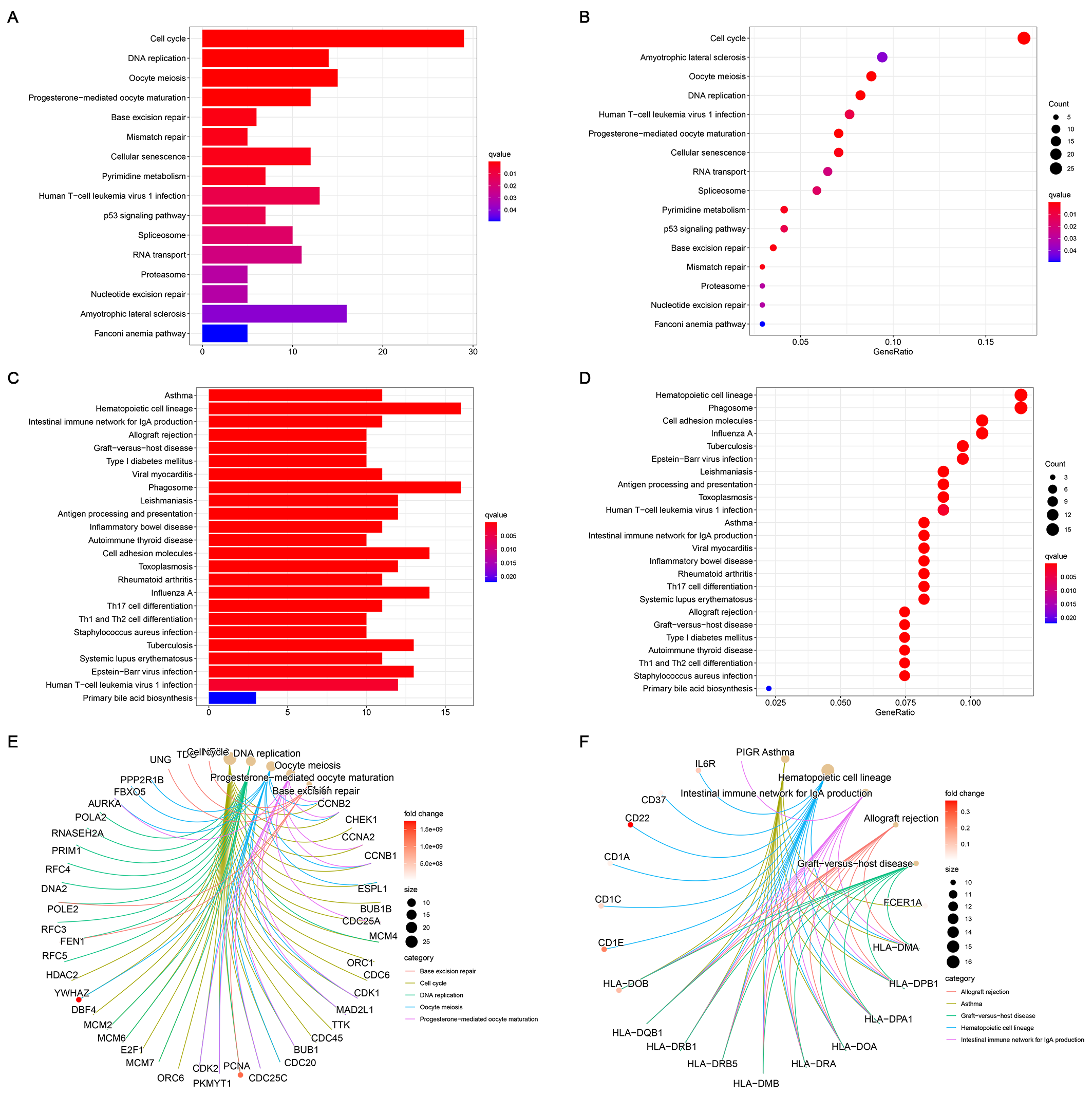

Supplement: Supplementary file 1 [file DataSheet1.ZIP › Supplementary Material/Supplementary Figures 3.tif]
